# Treatment of the Hair

**Published:** 1887-10

**Authors:** 


### TREATMENT OF THE HAIR.

A wineglassfull of aromatic spirits of ammonia, added to a basin full of water is very cleansing and refreshing. Care should be taken that it does not get into the eyes. The shampoo as given by the barber is too rough and vigorous, and the conglomeration he puts on the head afterward is anything but beneficial. While one performs daily ablutions of the face, hands and body, the head is generally left out. This should not be ; it is as necessary to wash the scalp as any other portion of the body. The hair should be brushed daily. Too much violence must be guarded against. It should be brushed gently in the direction in which it lies. A harsh brush should be used to cleanse the scalp of dust and dandruff, and then the hair shafts should be smoothed and polished by means of a softer brush. The scalp should receive a roseate glow. This insures quicker circulation in the follicle about the hair papilla, and hence the growth is invigorated. Hair tonics have the same effect upon the skin, viz : A stimulating effect upon the skin capillaries. Morning and night, before retiring is the best time for brushing the hair. Too hard brushing tends to produce dandruff. In brushing, the object is to cleanse it from extraneous materials, such as feathers, dust, dandruff, and concrete sebaceous material, which often oozes out upon the scalp, to make it smooth, and to bring truant hairs into the right place, and to set in harmony discordant filaments.

Friction polishes the hair as well as bandoline or ointment. The end we seek in building up a scanty hair crop is a proper amount of blood-supply through friction and hair tonics. The appended is an excellent hair tonic :

℞ Acid carbolic.................................ʒ ss ;

Tinct. nucis vom.............................ʒ ij ;

Tinct. cinchonæ rubræ.......................ʒ j ;

Tinct. cantharidis.............................℥ ss ;

Aq. cologniensis...............................

Ol. cocois......aa.......................q. s. ad ℥ iv. M

Apply once or twice a day to the scalp by means of a soft sponge. This will prevent the hair from falling out, if it does not produce a luxuriant crop.—*New York Medical Journal.*

